# Leadership for 2030 In Sight: defining the skills needed to drive change

**Published:** 2025-03-07

**Authors:** Anna McKeon

**Affiliations:** 1 Director of Capability Building: IAPB, London, UK.

**Read the first instalment in our six-part leadership series.**

**Figure:**
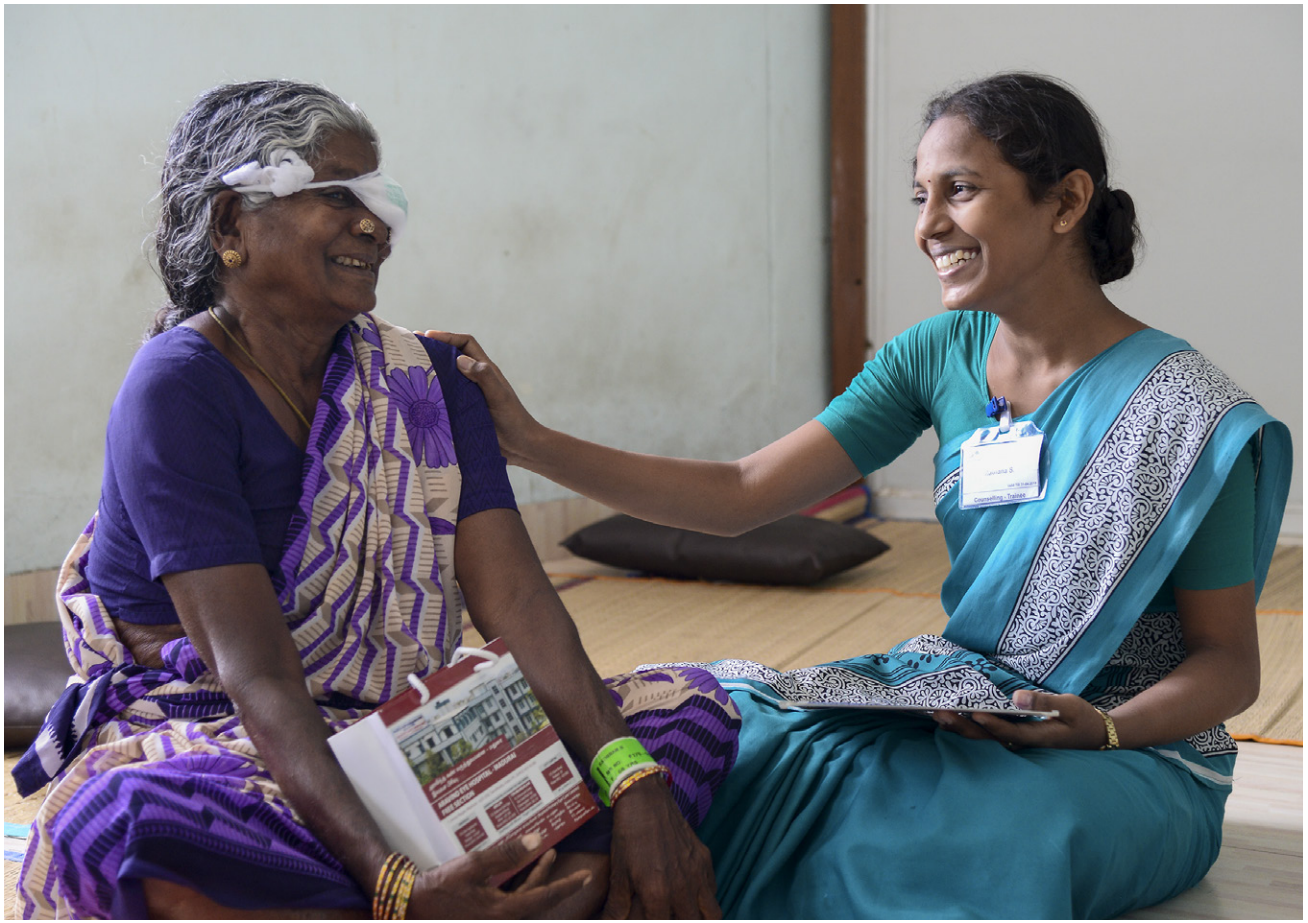
Compassionate leadership can make a big difference in eye health. INDIA

© RAMESH V RAMESH, COURTESY OF IAPB CC BY-NC-SA 4.0

The Lancet Global Commission on Global Eye Health: vision beyond 2020^1^ identified the need for a new kind of leadership in eye health: “The sector has produced many committed and effective leaders who excel at designing and managing eye health programmes. However, achieving progress across the development agenda will require leaving current approaches behind. A more innovative and adaptive approach that engages broad networks of diverse stakeholders is required. Leaders will need to be able to connect the whole system together.”

This is also reflected in the eye health sector strategy, **2030 In Sight**. If we are to end avoidable sight loss by 2030, we can't keep working in the same way and expecting different results. We need to do things differently. A change of approach, including different kinds of leadership skills, are needed.

But what does this kind of leadership look like in practice? Through interviews with experts in global eye health, and reviewing literature from social change movements, IAPB sought to define what leadership means in this context, and the key skills needed to help individuals drive transformative change.

## Understanding leadership: insights from the sector

Our interviews revealed a shared recognition that leadership in eye health is not about position or authority – it is about influence, vision, and the ability to mobilise collective action. Many interviewees emphasised that leadership is about “creating the conditions for change, not just directing others.” Another expert stated, “True leadership in eye health means helping people see their role in the bigger picture and equipping them to take action.”

Several participants highlighted that leadership is deeply contextual. What works in one setting may not work in another, and leaders must be attuned to cultural, political, and systemic differences. “You can't assume the same approach will work everywhere,” one respondent noted. “Listening and learning from the local context is key.”

“Leadership is deeply contextual. What works in one setting may not work in another, and leaders must be attuned to cultural, political, and systemic differences.”

Trust and collaboration were repeatedly identified as essential. One interviewee explained: “Building coalitions across disciplines is what moves the needle. We need leaders who don't just work within their own silos but actively bring others to the table.” The ability to build trust, communicate clearly, and engage diverse stakeholders is critical. Equally important is self-awareness: leaders must reflect on their own biases and assumptions to foster truly inclusive and equitable approaches.

## Drawing on existing leadership approaches

In defining leadership for 2030 In Sight, we drew on systems leadership and adaptive leadership approaches, because they best reflect the realities of leading change in complex dynamic environments.

**Systems leadership** provides a framework for understanding and influencing change at a systemic level. It encourages leaders to identify leverage points, foster collaboration across disciplines, and create enabling conditions for progress. As one expert noted, “Solving the eye health crisis is not about one organisation or one leader—it's about building a movement that brings together diverse voices and expertise.”

**Adaptive leadership** complements this by recognising that complex problems do not have fixed solutions. Leaders must be able to experiment, learn from failures, and adjust their approaches in response to evolving challenges. “Rigid leadership models don't work when policies shift and resources fluctuate,” one interviewee explained. “We need leaders who can remain focused on long-term goals while adapting to changing realities.”

## Defining leadership: key skills for impact

From this analysis, an approach to leadership emerged that emphasises systems leadership, strategic vision, collaboration, advocacy, and personal development. Leadership in eye health is not simply about executing plans, but about shaping the environment in which change can happen. It requires an understanding of how different factors – policy, funding, workforce development, and service delivery – interact to create challenges and opportunities.

Leadership for 2030 In Sight has been defined as: “a process through which an individual or organisation inspires and mobilises collective action to drive change within and across complex systems so that everyone, everywhere can access the eye care they need.”

This definition is complemented by five core skills that effective leaders in eye health need:

1. **Systems thinking**. Understanding the broader environment in which eye health operates, recognising interconnections, and identifying leverage points for change.
2. **Strategic vision**. Setting clear, ambitious goals that align with 2030 In Sight and mobilising stakeholders to achieve them.
3. **Collaboration and partnership building**. Working across sectors and disciplines to find shared solutions, fostering trust and mutual support.
4. **Advocacy and influencing**. Communicating compelling messages that drive policy change and secure resources for eye health initiatives.
5. **Personal leadership development**. Continuously learning, reflecting, and adapting to become more effective in different contexts.

## What next?

Leadership for 2030 In Sight is about more than individual skills – it is about shaping a collective movement for change. By developing these capabilities, practitioners at all levels can contribute to a future where everyone, everywhere, has access to quality eye care.

Over the coming issues of the *Community Eye Health Journal*, we will explore each of the five key leadership skills in more depth: systems thinking, strategic vision, collaboration and partnership building, advocacy and influencing, and personal leadership development.

**ACTION STEP**

To start preparing for the next article on systems thinking, take time to map the broader eye health landscape in your context. Identify key players, policies, and funding mechanisms that influence service delivery. Make use of the resources listed below:

- IAPB's eye health systems map (bit.ly/4cuFUb57)
- FSG's Guide to Actor Mapping (bit.ly/43TArbC)
- Systems mapping articles and tools from The Open University (bit.ly/42AvvGd).

**NEW SERIES Leadership for 2030 In Sight**

The sector strategy 2030 In Sight calls for a change of approach, and a different kind of leadership, in order to reach our ambition of ensuring accessible and affordable services for everyone, everywhere. We need to move away from vertical ways of working, siloed within health systems, and not only integrate eye care throughout health systems, but also demonstrate the role eye health has to play in other sectors and settings. Leaders need to be able to connect the whole system together.

IAPB has published the report **Leadership for 2030 In Sight**, which offers a definition of leadership for eye health and a set of critical skills needed to drive progress towards the goals of 2030 In Sight.

This series of articles on leadership will cover each of these skills, offering explanations, case studies, and inspiration. For anyone interested in continuing the conversation in more detail, please contact leadership@iapb.org. We'd be delighted to hear from you.

What to expect in the next five articles:

**Article 2. Systems leadership for sustainable change**

- Eye health as a complex system
- Shifting mindsets: leading as a systems activist
- Applying systems leadership in eye health (case study)

**Article 3. Creating a shared vision for change in a complex system**

- Understanding root causes and effective interventions
- Defining a bold, actionable vision
- Communicating for mobilisation (case study)

**Article 4. Collaboration and coalition building for systemic impact**

- Convening in complex systems
- Facilitation as a critical leadership skill
- Trust and relationships as the root of change (case study)

**Article 5. Advocacy and influencing for eye health transformation**

- What is advocacy? Making the case for systemic change
- Working with new and different actors
- Applying advocacy tools in practice (case study)

**Article 6. Systems leadership skills: developing others**

- Creating environments for leadership to flourish
- Psychological safety and inclusive leadership
